# The cardiovascular phenotype of childhood hypertension: a cardiac magnetic resonance study

**DOI:** 10.1007/s00247-019-04393-6

**Published:** 2019-05-03

**Authors:** Mun H. Cheang, Gregorz T. Kowalik, Michael A. Quail, Jennifer A. Steeden, Daljit Hothi, Kjell Tullus, Vivek Muthurangu

**Affiliations:** 1 Centre for Cardiovascular Imaging, University College London Institute of Cardiovascular Science and Great Ormond Street Hospital for Children, 30 Guilford Street London, London, WC1N 1EH UK; 2 Nephrology Department, Great Ormond Street Hospital, London, UK

**Keywords:** Aortic stiffness, Child, Chronic kidney disease, Hypertension, Magnetic resonance imaging, Renal artery stenosis, Systemic vascular resistance

## Abstract

**Background:**

The cardiovascular phenotype is poorly characterized in treated pediatric hypertension. Cardiovascular magnetic resonance imaging (MRI) can be used to better characterize both cardiac and vascular phenotype in children with hypertension.

**Objective:**

To use MRI to determine the cardiac and vascular phenotypes of different forms of treated hypertension and compare the results with those of healthy children.

**Materials and methods:**

Sixty children (15 with chronic renal disease with hypertension, 15 with renovascular hypertension, 15 with essential hypertension and 15 healthy subjects) underwent MRI with noninvasive blood pressure measurements. Cardiovascular parameters measured include systemic vascular resistance, total arterial compliance, left ventricular mass and volumetric data, ejection fraction and myocardial velocity. Between-group comparisons were used to investigate differences in the hypertension types.

**Results:**

Renal hypertension was associated with elevated vascular resistance (*P*≤0.007) and normal arterial compliance. Conversely, children with essential hypertension had normal resistance but increased compliance (*P*=0.001). Renovascular hypertension was associated with both increased resistance and compliance (*P*≤0.03). There was no difference in ventricular volumes, mass or cardiac output between groups. Children with renal hypertension also had lower systolic and diastolic myocardial velocities.

**Conclusion:**

Cardiovascular MRI may identify distinct vascular and cardiac phenotypes in different forms of treated childhood hypertension. Future studies are needed to investigate how this may inform further optimisation of blood pressure treatment in different types of hypertension.

**Electronic supplementary material:**

The online version of this article (10.1007/s00247-019-04393-6) contains supplementary material, which is available to authorized users.

## Introduction

Pediatric hypertension is a rare but serious condition, with a prevalence of approximately 3–6% [1, 2]. Chronic kidney disease, renal artery stenosis and essential hypertension are the most common causes of hypertension in children [3, 4]. Although elevated blood pressure is a common feature, it is unclear which components of vascular function (resistance, compliance or cardiac output) are abnormal in each condition. This is because comprehensive vascular phenotyping is difficult to perform using conventional noninvasive techniques. Recently, cardiovascular magnetic resonance imaging (MRI) has been combined with noninvasive pressure measurements to accurately evaluate vascular function in children. Using this technique, it has been shown that children with renal disease have raised systemic vascular resistance compared to healthy children [5]. We believe this approach could be used to explore different vascular phenotypes in pediatric hypertension.

Another benefit of MRI is that it enables simultaneous cardiac phenotyping. This includes reference standard assessment of left ventricular mass and volumes [6], as well as novel evaluation of cardiac timings, mitral inflow and myocardial velocities [7, 8]. This ability is useful as the myocardial response to hypertension may also vary depending on the underlying cause.

The aim of this study is to use to MRI to determine the cardiac and vascular phenotypes of children with renal disease and hypertension, renovascular disease and essential hypertension and to compare the results with those of healthy children.

## Materials and methods

### Study population

The study population consisted of 60 children: 15 children with renal parenchymal disease and hypertension (renal), 15 children with renal artery stenosis associated hypertension (renovascular), 15 children with idiopathic essential hypertension and 15 healthy controls. Only patients with stable, treated hypertension were recruited. Exclusion criteria were: 1) age <7 or >18 years, 2) congenital structural heart disease or primary myocardial disease, 3) significant renal impairment in renovascular or essential hypertension, 4) active vasculitis, 5) cardiac arrhythmia precluding the use of cardiac gated MRI sequences, 6) medical devices precluding MRI, 7) other causes of secondary hypertension, and 8) current or previous renal replacement therapy. The exclusion criteria were selected to ensure that the study groups did not have any other condition that may account for cardiovascular abnormalities.

This study was approved by the United Kingdom national research ethics service (National Research Ethics Service Committee London, London Bridge, REC reference: 15/LO/0213). Informed parental consent and patient assent were obtained from all participants. Patients were consecutively recruited from hypertension and renal clinics in Great Ormond Street Hospital over 24 months.

The renal group was a subset of a previously examined group of pediatric chronic kidney disease patients who had been phenotyped using MRI [5]. Only renal patients with a confirmed diagnosis of hypertension were included in this study.

In the renovascular group, renal artery stenosis was confirmed with a combination of noninvasive and invasive investigations [9]. All renovascular children received the optimal treatment plan as recommended following discussion by a multidisciplinary clinical team.

The essential hypertension group was clinically evaluated to exclude other secondary causes of hypertension and treated according to published recommendations [10].

### Imaging protocol

Imaging was performed on a 1.5-T MRI system (Avanto; Siemens Medical Solutions, Erlangen, Germany). All MRI data were processed using in-house plug-ins developed for open-source DICOM (Digital Imaging and Communications in Medicine) software OsiriX [11] (the OsiriX Foundation, Geneva, Switzerland) by the same individual (M.H.C., with >5 years’ experience in cardiac MRI), who was masked to the subject. No gadolinium was given. Summary details of MRI sequence scan parameters are summarized below.

### Cardiac volumes and mass

Left ventricular volumes were assessed using short axis multi-slice free-breathing real-time steady-state free precision sequence [12]. Real-time radial k-t SENSE steady-state free precision sequence parameters were field of view: approximately 350 mm, matrix: 128×128, voxel size: approximately 2.7×2.7×8 mm, echo time (TE)/repetition time (TR): approximately 1.1/2.2 ms, flip angle: 40°, acceleration factor: 8, and temporal resolution: approximately 36 ms. Processing of the left ventricular short axis data was performed as previously described, enabling measurement of end-diastolic volume, end-systolic volume, stroke volume, ejection fraction and left ventricular mass. All ventricular volumes were indexed to body surface area. The effect of body size on the left ventricular mass was controlled by indexing to height to the power of 2.7 [13]. The right and left atrial areas in diastole were measured from a four-chamber view and indexed to body surface area.

### Cardiac timing and inflow velocities

Left ventricular outflow tract and mitral inflow velocities were assessed with a free-breathing high temporal resolution real-time phase contrast MR sequence [7]. Real-time UNFOLD-SENSE spiral phase contrast MR sequence parameters were field of view: approximately 450 mm, matrix: 128×128, voxel size: approximately 3.5×3.5×7 mm, TE/TR: approximately 1.97/ 7.41 ms, flip angle: 20°, maximum measurable velocity range: 150 m/s, acceleration factor: 10, and temporal resolution: approximately 15 ms. The mitral valve orifice and left ventricular outflow tract were manually segmented from the resultant inflow and outflow curves to obtain the isovolumic relaxation time, isovolumic contraction time and ejection time as previously described [7]. Peak early and atrial diastolic velocities were also measured from the inflow curves.

### Myocardial velocities

Left ventricular myocardial velocities were measured using a previously validated free-breathing tissue phase mapping sequence planned in the mid left ventricular short axis view [8]. Retrospectively gated golden-angle spiral tissue phase mapping sequence details were field of view: approximately 400 mm, matrix: 192×192, voxel size: approximately 2.1×2.1×8 mm, TE/TR: approximately 3.51/ 11.7 ms, flip angle: 15°, respiratory navigation efficiency: 30%, scan time: approximately 7–8 min, and temporal resolution: approximately 23 ms. Each acquisition produced a magnitude image and three phase images (in the x-, y- and z-direction). The radial velocity was calculated by transforming the x- and y-direction velocities to an internal polar coordinate system using the left ventricular center of mass as a reference point. The longitudinal velocity was taken as velocity in the z-direction. Global radial and longitudinal left ventricular velocities were calculated by averaging velocities across the segmented left ventricular slice for a given direction. The magnitude of the peak systolic and early diastolic velocities was measured from the velocity time curves.

### Aortic flow

**Fig. 1:**
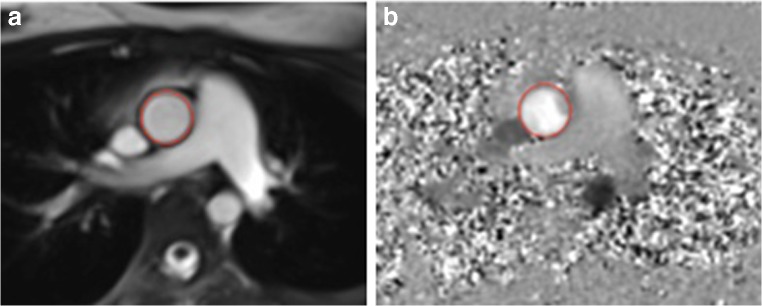
Phase contrast MR images acquired at the level of the ascending aorta of a 15 year old boy. **a** Magnitude image, (**b**) phase image. The region of interest (*circles*) traces the outline of the cross-sectional aorta area throughout all phases of the cardiac cycle to obtain accurate cardiac output for calculating systemic vascular resistance and total arterial compliance

Aortic flow assessment was performed using a breath-held retrospectively gated phase contrast MR technique just above the sinotubular junction (Fig. 1) [14]. Retrospectively gated spiral SENSE phase contrast MR sequence parameters were field of view: approximately 400 mm, matrix: 256×256, voxel size: approximately 1.6×1.6×5 mm, TE/TR: approximately 2.1/8.0 ms, flip angle: 25°, acceleration factor: 3, breath-hold time: 4–8 s, and temporal resolution: approximately 32 ms. The aorta was segmented to produce a flow curve from which stroke volume and cardiac output were derived (Fig. 1). The maximum and minimum cross-sectional areas of the ascending aorta over the cardiac cycle were also recorded.

### Blood pressure measurement

Brachial systolic, diastolic and mean arterial blood pressures were measured from the right arm in all subjects using an MRI compatible oscillometric sphygmomanometer (Datex Ohmeda; General Electric, Boston, USA) during acquisition of MRI data. This enabled an optimum combination of blood pressure and flow data for calculating vascular indices. All blood pressure measurements were acquired with an appropriately sized arm cuff after the subject had been prone in the scanner for at least 10 min. Pulse pressure was the difference between systolic and diastolic blood pressure.

### Measures of vascular characteristics

**Fig. 2:**
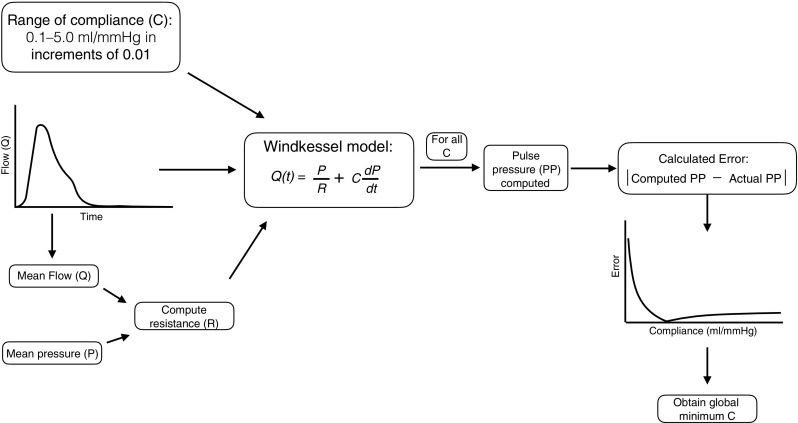
Algorithm for calculating compliance. *C* total arterial compliance, *P* pressure, *PP* pulse pressure, *Q* flow, *Q(t)* flow over time, *R* resistance, *t* time

Total systemic vascular resistance was calculated by dividing mean blood pressure by cardiac output [15]. Total arterial compliance was calculated using a two-element Windkessel model (Fig. 2) described previously [15]. Briefly, aortic flow curves were inputted into the model with measured systemic vascular resistance. The compliance was tuned so that pulse pressure generated by the model equaled measured pulse pressure (Fig. 2) [15]. Local arterial stiffness was assessed by calculating ascending aortic compliance = (difference of maximum and minimum aortic cross-sectional area)/pulse pressure [16].

### Statistics

Statistical analyses were performed using Stata 13 (StataCorp, College Station, Texas, USA). A *P*-value<0.05 was considered statistically significant. Data were examined for normality using the Shapiro-Wilk normality test and non-normally distributed data were transformed using a zero-skewness log transform to ensure normal distribution before analysis. Descriptive statistics were expressed as mean (±standard deviation) or geometric mean (±geometric standard deviation) if data were log transformed. The chi-square test was used to determine if there were differences in the use of hypertensive medications among the three hypertension groups and in gender distribution across all groups. Group differences were assessed using analysis of variance (ANOVA). Levene’s test was used to assess for homogeneity of variances across the groups and Welch’s correction was applied for nonhomogeneous variance. Post hoc pairwise comparison was performed for significant results on ANOVA, using Duncan’s method to adjust for potential Type 1 errors. Age- and gender-adjusted ANOVA tests were also performed for indices with significant group differences. To determine if group differences in myocardial velocities were due to associated cardiac and vascular factors, Pearson’s correlations were first performed between myocardial velocities (with significant group differences) and possible variables. ANOVA tests for these myocardial velocities were then repeated after adjustment for any significantly correlated variables (as well as age and gender).

## Results

### Study population

The renal cohort consisted of pre-dialysis patients in chronic kidney disease stages 2–4 (Stage 2: 5 children, Stage 3: 7 children, Stage 4: 3 children) with stable renal function. Mean estimated glomerular filtration rate was 53±25 ml/min/1.73 m^2^. The causes of renal impairment were congenital abnormalities of the kidney and urinary tract (*n*=8), renal dysplasia (*n*=3), hemolytic uremic syndrome (*n*=2), glomerulonephritis (*n*=1) and tubulointerstitial disease (*n*=1). All patients were diagnosed with hypertension and received hypertensive treatment (Table 1). Seven (47%) were taking two or more medications.

**Table 1:** Demographics and baseline characteristics of study population

	Healthy controls *n*=15	Renal hypertension *n*=15	Renovascular hypertension *n*=15	Essential hypertension *n*=15	*P*-value
Age (years)^a^	12±1.3	13±1.3	12±1.3	14±1.3	0.16
Gender (% male)^b^	53%	60%	73%	73%	0.58
Height (cm)	156±14	154±18	156±18	164±14	0.32
Weight (kg)^a^	50±1.3	44±1.4	48±1.4^c^	68±1.4^d^	0.003
Body mass index (kg/m^2^)	21±2.3	19±3.6	20±3.6^c^	26±6.2^d^	0.010^e^
Body surface area (m^2^)	1.5±0.24	1.4±0.3	1.5±0.35^c^	1.8±0.34^d^	0.006
Heart rate (beats per minute)^a^	73±1.2	78±1.3	73±1.2	83±1.2	0.19
Medications (%)^b^					
*A*ngiotensin converting enzyme or angiotensin 2 receptor inhibitor		67%	20%	53%	0.03
Beta-blocker		40%	33%	13%	0.25
Calcium channel blocker		47%	40%	53%	0.77

^a^Logarithmic transformation was applied

^b^A chi-square test was performed. A chi-square test was performed between all four groups for gender. A chi-square test was performed between the three hypertensive groups for medications

^c^*P* value<0.05 when renovascular hypertension is compared to hypertension

^d^*P*<0.05 when essential hypertension is compared with controls; *P*<0.05 when hypertension is compared with renal hypertension

^e^ANOVA Welch (W) test was used

All renovascular patients had objective evidence of renal artery stenosis. Of those, 50% (*n*=6) had bilateral renal artery stenosis. The majority (*n*=10) were treated initially with angioplasty, while the remaining patients received ethanol embolization of a renal artery (*n*=1), unilateral nephrectomy (*n*=1), surgical revascularization (*n*=1) or medical management (*n*=2). None was awaiting further invasive treatment. All patients had been deemed by their clinicians to have controlled hypertension with clinically stable blood pressure. The majority of renovascular children (*n*=9) continue to receive hypertension medication (Table 1). Of these, five were taking two or more drugs. Forty percent (*n*=6) were no longer on hypertension medication.

All essential hypertension patients had a diagnosis of idiopathic hypertension and had stable treated blood pressure when last reviewed in the outpatient clinic (Table 2). Five patients were on two hypertensive medications.

**Table 2:** Vascular phenotype of study population

	Healthy controls *n*=15	Renal hypertension *n*=15	Renovascular hypertension *n*=15	Essential hypertension *n*=15	*P*-value
Systolic blood pressure (mmhg)	103±11	125±13^b^	122±8.4^c^	128±12^e^	<0.001
Diastolic blood pressure (mmhg)	52±6.6	75±10^b^	63±12^c,d^	67±13^e,f^	<0.001
Mean blood pressure (mmhg)	74±5.9	95±8.7^b^	88±8.6^c,d^	92±11^e^	<0.001
Pulse pressure (mmhg)	51±12	50±12	59±13	62±9.5^e,f^	0.016
Systolic blood pressure percentile	25±2.5	76±1.6^b^	80±1.3^c^	81±1.3^e^	<0.001^g^
Diastolic blood pressure percentile	22±14	77±19^b^	50±31^c,d^	52±33^e,f^	<0.001^g^
Cardiac output (l/min/m^2^)^a^	3.7±1.2	3.6±1.2	3.6±1.2	3.8±1.2	0.73
Systemic vascular resistance (WU.m^2^)	20±3.6	27±5.3^b^	25±5.5^c^	24±4.3^e^	0.003
Total arterial compliance (ml/mmHg. m^2^)	0.59±0.14	0.55±0.11	0.5±0.11^c^	0.44±0.06^e,f^	0.002
Ascending aortic compliance (%mmHg^−1^.10^2^)^a^	3.2±1.5	2.7±1.5	1.7±1.5^c,d^	2.2±1.5^e^	<0.001

^a^Logarithmic transformation was applied

^b^*P*-value<0.05 when renal hypertension is compared with controls

^c^*P*-value<0.05 when renovascular hypertension is compared with controls

^d^*P*-value<0.05 when renovascular hypertension is compared with renal hypertension

^e^*P*-value<0.05 when essential hypertension is compared with controls

^f^*P*-value<0.05 when essential hypertension is compared with renal hypertension

^g^ANOVA Welch (W) test was used

Although there was no difference in age or gender distribution (Table 1), weight was significantly different (*P*<0.001) among groups, even after adjusting for age and gender. On pairwise comparison, the essential hypertension patients were significantly heavier compared to the renovascular and renal groups (*P*≤0.017).

### Differences in blood pressure

Blood pressure data are shown in Table 2. Systolic blood pressure was higher in all hypertensive groups compared to the controls (*P*<0.001), but there were no differences among the three hypertension groups (*P*>0.11). The systolic blood pressure percentiles were also higher in all the hypertension groups compared to controls (*P*<0.001). Nineteen patients (four renovascular, eight renal, seven essential hypertension) had uncontrolled systolic hypertension (systolic blood pressure >90 percentile).

Diastolic blood pressure was higher in the hypertension groups compared to the controls (*P*≤0.008). In addition, children with renal disease had higher diastolic blood pressure than both the renovascular (*P*=0.004) and essential hypertensive (*P*=0.035) groups. A similar pattern was seen in diastolic blood pressure percentiles, with six patients (no renovascular, four renal, two essential hypertension) having uncontrolled diastolic hypertension (diastolic blood pressure >90 percentile) at the time of the MRI scan.

The mean blood pressure was higher in all hypertension groups compared to controls (*P*<0.001). Furthermore, the renal group had a significantly higher mean blood pressure than the renovascular group (*P*=0.027). Conversely, pulse pressure was only elevated in the essential hypertension children compared to the controls (*P*=0.019). The pulse pressure was also significantly higher in the essential hypertension patients compared to the renal patients (*P*=0.012). These differences remained broadly present after controlling for age and gender.

### Vascular properties

Vascular measures are shown in Table 2. Systemic vascular resistance was higher in all hypertension groups compared to the controls (*P*≤0.028) and there were no significant differences between the hypertension groups (*P*>0.15). However, after correcting for age and gender, the systemic vascular resistance in the essential hypertension group was no longer significantly different from the controls (*P*=0.13).

Total arterial compliance was significantly lower in the essential hypertension and renovascular groups compared to the controls (*P*≤0.026) and in the essential hypertension group compared to the renal children (*P*=0.01). Local ascending aortic compliance was also lower in the essential hypertension and renovascular children compared to the controls (*P*≤0.018). Furthermore, the renovascular children had significantly lower ascending aortic compliance compared to the renal patients (*P*=0.005). Differences in total and local compliance remained after correcting for age and gender. The cardiac output was similar in all groups (Table 2).

### Cardiac structure and global function

Left ventricular volumes and function are shown in Table 3. There were no significant differences in the left ventricular volumes or function among the groups, including after age and gender correction. There was also no difference in height indexed left ventricular mass among the groups.

**Table 3:** Assessment of myocardial structure and left ventricular systolic and diastolic function

	Healthy controls *n*=15	Renal hypertension *n*=15	Renovascular hypertension *n*=15	Essential hypertension *n*=15	*P*-value
End-diastolic volume (ml/m^2^)	74±9.4	69±12	69±9.3	70±9.8	0.45
End-systolic volume (ml/m^2^)	23±4.2	22±5.9	19±7.8	22±5.3	0.22
Stroke volume (ml/m^2^)	51±6.4	47±9.2	50±7.1	47±5.4	0.33
Indexed left ventricular mass (g/m^2.7^)	23±2.9	23±6.3	25±5.4	27±6.7	0.14
Right atrial area (cm^2^/m^2^)	12±1.6	11±1.9	11±2.1	11±1.4	0.06
Left atrial area (cm^2^/m^2^)^a^	12±1.3	12±1.2	12±1.1	10±1.1^b^	0.04
Ejection fraction (%)	69±3.4	68±6.2	73±9.1	68±3.8	0.32^c^
Isovolumic relaxation time (ms)	64±11	70±12	67±11	69±13	0.51
Isovolumic contraction time (ms)	47±15	41±14	54±27	49±22	0.41^c^
Early diastolic (E) mitral flow (ml)	47±9.6	52±9.8	57±11	48±9.8	0.05
Late diastolic (A) mitral flow (ml)	19±4.2	21±6.6	25±6.6	23±5.7	0.11
E/A ratio^a^	2.5±1.2	2.5±1.6	2.3±1.4	2.2±1.3	0.60
Ejection time (ms)	278±19	277±13	276±22	266±15	0.27

^a^Logarithmic transformation was applied

^b^*P*<0.05 when essential hypertension is compared with controls, *P*<0.05 when essential hypertension is compared with renal hypertension, *P*<0.05 when renovascular hypertension is compared to essential hypertension

^c^ANOVA Welch (W) test was used

Mitral valve inflow and cardiac timing measures are shown in Table 3. There were no significant group differences in early or late mitral inflow velocities, early to late mitral velocities ratio, or isovolumic times, including after correcting for age and gender.

### Myocardial velocities

Myocardial velocities are shown in Table 4. There were no differences between the radial peak systolic velocity in the hypertension groups and the controls (*P*>0.12). However, renal and renovascular children did have lower radial peak systolic velocities compared to the essential hypertension children (*P*=0.009 and *P*=0.047, respectively). After adjustments for age and gender, only renal children had lower radial peak systolic velocities than both the controls and the essential hypertension children (*P*=0.027 and *P*=0.029, respectively). Radial peak systolic velocity was negatively correlated with systemic vascular resistance (r=−0.3, *P*=0.022) and positively correlated with left ventricular mass (r=0.26, *P*=0.049). Radial peak systolic velocity did not correlate with other hemodynamic metrics (*P*>0.15). Group differences in radial peak systolic velocities were abolished after adjusting for systemic vascular resistance (*P*=0.41), but they remained after adjustment for left ventricular mass (*P*=0.043). There were no group differences in longitudinal peak systolic velocity.

**Table 4:** Assessment of myocardial velocities

	Healthy controls *n*=15	Renal hypertension *n*=15	Renovascular hypertension *n*=15	Essential hypertension *n*=15	*P*-value
Radial peak systolic myocardial velocity (cm/s)	2.7±0.41	2.5±0.42	2.6±0.27	2.9±0.3^d^	0.035
Radial early diastolic velocity (cm/s)	4±0.85	3.3±0.65^b^	4±0.7^c^	3.8±0.39	0.018
Longitudinal peak systolic myocardial velocity (cm/s)^a^	4±1.3	3.3±1.4	3.3±1.5	3.7±1.2	0.31
Long early diastolic velocity (cm/s)	7.5±1.8	7.6±2.2	7.3±1.9	9.5±1.7^d,e^	0.01

^a^Logarithmic transformation was applied

^b^*P*<0.05 when renal hypertension is compared with controls

^c^*P*<0.05 when renovascular hypertension is compared with renal hypertension

^d^*P*<0.05 when renovascular hypertension is compared to essential hypertension, *P*<0.05 when essential hypertension is compared with renal hypertension

^e^*P*-value<0.05 when essential hypertension compared with controls

The radial early diastolic velocity was significantly reduced in the renal group compared to both the controls (*P*=0.009) and the renovascular children (*P*=0.009). These differences remained after correcting for the influence of age and gender. Radial early diastolic velocity only correlated with diastolic blood pressure (r=0.43, *P*<0.001), mean blood pressure (r=0.31, *P*=0.017), systemic vascular resistance (r=0.47, *P*<0.001) and radial peak systolic (r=0.53, *P*<0.001). Group differences remained after adjusting for systemic vascular resistance (*P*=0.045) or radial peak systolic (*P*=0.022), but were abolished after adjusting for diastolic (*P*=0.25) or mean blood pressure (*P*=0.11).

The longitudinal early diastolic velocity was significantly increased in the essential hypertension group compared to the controls (*P*=0.009), the renovascular (*P*=0.006) and the renal children (*P*=0.009). These differences remained after correcting for the influence of age and gender. The longitudinal early diastolic velocity was only correlated with the left ventricular mass (r=0.30, *P*=0.02). After adjusting for the left ventricular mass, there was only a trend for group differences (*P*=0.055).

## Discussion

This study investigated the cardiovascular phenotype in different forms of treated hypertension in children with MRI. Our findings were:

- Systemic vascular resistance was elevated in children with renal and renovascular disease, but was normal in essential hypertension after adjusting for age and gender.
- Arterial stiffness (i.e. lower total arterial compliance and ascending aortic compliance) was higher in essential hypertension and renovascular, but was normal in renal disease.
- Children with renal disease had reduced radial systolic and diastolic function (lower peak systolic and early diastolic myocardial velocities, respectively) compared to the controls and the other hypertension groups.

### Vascular phenotype

It is increasingly recognised that differences in the pattern of hypertension is associated with differential mortality risk [17]. Systolic and diastolic hypertension in young adults, which is associated with an elevated systemic vascular resistance, have been shown to be associated with the highest risk when compared to other patterns of hypertension such as isolated systolic hypertension [17]. Thus, the pattern of hypertension is closely related to the vascular phenotype and may provide valuable prognostic information [18].

In this study, the blood pressure phenotype in the three hypertension groups was slightly different, despite having similar systolic blood pressure. Specifically, the renal patients had higher diastolic blood pressure and mean blood pressure, while the essential hypertension group had greater pulse pressure. These findings are in keeping with previous large studies of blood pressure in these populations [5, 19] and suggest differing vascular abnormalities. However, it is not possible to definitively characterize vascular phenotype using this information because of the important role of cardiac output in determining blood pressure. Therefore, we combined blood pressure measurements with cardiac MRI to assess cardiac output and calculate systemic vascular resistance and total arterial compliance. This allowed full characterization of vascular phenotype and ruled out the possibility that blood pressure differences were simply due to differences in cardiac output.

We demonstrated that hypertensive renal disease was associated with increased systemic vascular resistance, whilst essential hypertension was characterized by decreased total arterial compliance and ascending aortic compliance. Children with renovascular hypertension fell somewhere in between these two groups. These findings are in keeping with the blood pressure phenotypes of elevated pulse pressure in the essential hypertension patients and elevated mean blood pressure in the renal disease patients. However, it should be noted that this is a treated population and untreated children may have different or more marked vascular abnormalities. Consequently, extrapolating these findings may be premature, and further studies are required. Nevertheless, our findings suggest that treated children cannot be considered to have normal vascular phenotype.

Raised systemic vascular resistance is known to occur in renal parenchymal disease [20] and possible causes include persistent abnormal renin-angiotensin stimulation [21], sympathetic overdrive [22] and reduced endothelial nitric oxide bioavailability [23]. Conversely, reduced total arterial compliance and increased pulse pressure are recognised to be central to the development of essential hypertension [24–26]. Our data further suggest that even after treatment, the essential hypertension children continue to have increased vessel stiffness.

Renal artery stenosis results in renal ischaemia, which in turn leads to wide-ranging maladaptive neurohumoral and vascular responses. Vasoconstriction is primarily mediated through renin-angiotensin-aldosterone axis activation and increased sympathetic activity in renovascular hypertension [27, 28]. Thus, renovascular children would be expected to have significantly elevated systemic vascular resistance before invasive treatment [27, 28].

Our results indicate that this does not completely normalize after therapy. Furthermore, the increased arterial stiffness in renovascular cases suggests that any abnormal vascular remodelling that may be the result of high pre-treatment blood pressure appears to persist despite successful therapy.

The fact that these treated patients continue to have raised blood pressure is important as even mildly elevated blood pressure confers additional cardiovascular risk, particularly in those burdened from a young age [17]. Thus, further treatment escalation may be appropriate and better understanding of the underlying pathophysiology might help determine success. For instance, children with renal disease may benefit from more aggressive vasodilation with more use of calcium channel blockers. Conversely, essential hypertension patients may be better treated with newer therapies such as neprilysin inhibitors (e.g., Sacubitril/Valsartan) that target aortic stiffness [18].

### Cardiac phenotype

One of the major benefits of using MRI to assess these children is the ability to comprehensively evaluate the myocardial response to elevated blood pressure. In this study, conventional metrics of left ventricular systolic and diastolic function were normal. Furthermore, the left ventricular mass was not increased in the hypertension groups. The lack of significant changes in the left ventricular structure and global function is probably related to the fact that this was a treated population. However, we did find abnormal myocardial velocities with both radial peak systolic and early diastolic velocities being different amongst the groups. We also showed that radial peak systolic and early diastolic velocities correlated with measures of afterload, particularly systemic vascular resistance (peak systolic and early diastolic velocities) and diastolic blood pressure (early diastolic velocities). This is in keeping with the well-recognized association between afterload and both systolic and diastolic function [29]. Interestingly, there were no group differences in radial peak systolic or early diastolic velocities in models adjusted for systemic vascular resistance (for peak systolic velocity) or diastolic blood pressure (for early diastolic velocity). This suggests that differences in myocardial velocities in the different groups of pediatric hypertension are at least partly explained by differences in vascular phenotype. However, our findings do not preclude direct myocardial disease in some of these patients (particularly the renal children) due to the small patient population.

There is also evidence from our study that radial early diastolic velocity correlates with radial peak systolic velocity. This is in keeping with the known association between systolic and diastolic function [30], but did not explain group differences in radial early diastolic velocities. A surprising finding of this study was that patients with essential hypertension appeared to have higher longitudinal early diastolic velocities. We think this is due to the higher bulk motion of the heart in essential hypertension patients, rather than better myocardial relaxation. Although it is possible to correct for radial bulk motion using short axis tissue phase mapping, it is not possible to correct for longitudinal bulk motion. This is a limitation of this technique and in the future, four-chamber or long axis approaches should be considered.

### Limitations

The main limitation of this study is the small number of patients in each group. The main reason for the small numbers was the difficulty in recruiting renal artery stenosis patients. Renal artery stenosis is a relatively rare condition and may be associated with other comorbidities. As other conditions such as significant midaortic syndrome may have potential confounding effects, it was important that only a “pure” population of renal artery stenosis was included in the study. Hence, it was not possible to recruit a larger number of renal artery stenosis children from a single-centre study. However, in spite of that, we were able to demonstrate significant differences in cardiovascular structure and function among the groups using MRI. Nevertheless, larger multicentre studies are required to properly confirm our results. The relatively small study numbers also precluded the inclusion of additional potential confounding factors into the ANOVA models such as differences in hypertensive treatment and the length of treatment among the groups. Variation in current medication therapy may contribute to differences in vascular phenotype among the groups. Future studies will need to consider multicentre recruitment to increase the power of the study population to adjust for the potential effects of different hypertensive treatment.

Another important limitation was that the majority of patients were being actively treated with anti-hypertensive medication. This was because of difficulties in recruiting patients before they began therapy. In particular, most patients were referred to our center having already been diagnosed with hypertension and started on therapy. This also made it difficult to ascertain the length of diagnosis and therapy. Thus, we cannot make firm conclusions about the differences in cardiac and vascular phenotype in treatment-naïve patients or the response to treatment in the different groups. In particular, we are not powered to investigate the effect of length of treatment on vascular or cardiac phenotype. Nonetheless, we were able to demonstrate persistent vascular changes associated with renal disease and hypertension despite optimal clinical treatment in this population. Future studies should consider a prospective evaluation of the effects of anti-hypertensive therapy in treatment-naïve children, including investigation of the time course of any improvements.

A final limitation is that the supine resting position may also be associated with elevated systemic vascular resistance [31]. However, as the method for systemic vascular resistance assessment was applied identically across the entire study cohort, the intergroup differences demonstrated in the study are likely to be significant.

## Conclusion

We have shown that there are differences in vascular and cardiac phenotype among the different types of treated hypertension in childhood. Future studies in treatment naïve children are needed to better understand if these different phenotypes have any prognostic implications or could be used to optimise blood pressure control.

## Electronic supplementary material

ESM 1 (DOCX 58 kb)
