# Supplementary material for: The cardiovascular phenotype of childhood hypertension: a cardiac magnetic resonance study
Source: Pediatr Radiol. 2019 May 3;49(6):727–36. doi: 10.1007/s00247-019-04393-6 (PMC6614159; doi:10.1007/s00247-019-04393-6)
Supplement: Supplementary file 1 — (DOCX 58 kb) [file 247_2019_4393_MOESM1_ESM.docx]

**Online supplementary document**

**Online Supplement Table 1: Associated co-morbidities of study population**

|  | Associated co-morbidities | No. of patients(%) |
| --- | --- | --- |
| Hypertensive Chronic | Asthma | 2 (13%) |
| Kidney Disease | Mild cerebral palsy | 1 (7%) |
| Renovascular | Fibromuscular dysplasia | 1 (7%) |
| Hypertension | Previous embolic stroke | 1 (7%) |
|  | Asthma | 3 (20%) |
|  | Neurofibromatosis | 3 (20%) |
|  | Coeliac disease | 1 (7%) |
| Essential Hypertension | Previous intracerebral bleed | 1 (7%) |
|  | C6 complement deficiency | 1 (7%) |
|  | Asthma | 1 (7%) |
|  | G6PD deficiency | 1 (7%) |
|  | Idiopathic focal epilepsy | 1 (7%) |
